# Genetic characterization reveals evidence for an association between water contamination and zoonotic transmission of a *Cryptosporidium* sp. from dairy cattle in West Bengal, India

**DOI:** 10.1016/j.fawpar.2019.e00064

**Published:** 2019-08-22

**Authors:** Koushik Das, Lakshmi V. Nair, Ajanta Ghosal, Sanjib Kumar Sardar, Shanta Dutta, Sandipan Ganguly

**Affiliations:** a Division of Parasitology, National Institute of Cholera and Enteric Diseases, P-33 CIT Road, Scheme XM, Beliaghata, Kolkata 700010, West Bengal, India; b Department of Microbiology, All India Institute of Hygiene and Public Health, 110 Chittaranjan Avenue, Kolkata 700073, West Bengal, India

**Keywords:** *Cryptosporidium* sp., Dairy, Zoonotic, 18SrRNA, Association, Genetic recombination

## Abstract

*Cryptosporidium* sp. is an enteric parasite with zoonotic potential, and can infect a wide range of vertebrates, including human. Determining the source of infection and the mode of transmission in a new endemic region is crucial for the control of cryptosporidiosis. In the present study, we have assessed the importance of dairy cattle as a potential source of *Cryptosporidium* infection for humans in a newly recognized endemic region. *Cryptosporidium* isolates from dairy calves, humans (farm workers) and nearby water bodies were genetically characterized based on *18SrRNA* and *hsp70* genes. A high incidence of *Cryptosporidium* infection was identified in our study region. This finding is of public health concern. *Cryptosporidium ryanae* rather than *Cryptosporidium parvum* has been identified as the most prevalent infecting species in the study region. Infections were associated with clinical symptoms of infected animals. An incomplete linkage disequilibrium (LD) value with potential recombination events at *18SrRNA* locus were identified for the first time in *C. ryanae*, which was previously reported as a clonal population. Phylogenetic analysis revealed the presence of identical genotypes of a *Cryptosporidium* sp. from dairy calves, farm workers and nearby water bodies and indicates an association between water contamination and zoonotic transmission of Cryptosporidiosis in our study region.

## Highlights

• Increased occurrence of *Cryptosporidium* infection was identified.
• *C. ryanae* rather than *C. parvum* was identified as most prevalent infecting species.
• Infection has significant association with age, sex and symptoms of infected animal.
• Evidence of genetic recombination was identified in *Cryptosporidium* population.
• Phylogenetic analysis indicates water-borne infection and zoonotic transmission.

## 1 Introduction

*Cryptosporidium* is a coccidian parasite infecting a wide range of vertebrates, including humans. Disease transmission typically occurs via fecal-oral route following either direct contact with contaminated fecal samples from an infected host or indirectly through contaminated water or food. Cattle is a major reservoir for *Cryptosporidium* spp. (Zhao et al., 2014). Individuals in close proximity with infected animals are at a high risk of acquiring cryptosporidiosis, especially cattle handlers, veterinarians and others working in low hygiene areas. The disease is usually self-limiting in immunocompetent individuals, but can be life-threatening or fatal among malnourished and immunocompromised patients (Steeb et al., 1987; Kurniawan et al., 2013). Unlike for other causative agents of infectious enteritis, there are no widely available, effective vaccines or drug-based intervention strategies for *Cryptosporidium*, and hence control mainly focuses on disease prevention (Jex et al., 2011). Identification of infection reservoirs and mode of transmission are crucial to prevent transmission and control disease. Genetic characterization of *Cryptosporidium* isolates from infected hosts as well as from environmental sources may be important for determining the mode of transmission in a new endemic region (Xiao, 2010). We have been engaged in a systematic surveillance study in West Bengal, India to determine the detection rate of common enteric parasites. *Cryptosporidium* spp. have been identified as one of the common infecting parasite. The objectives of the present study were to identify the infection reservoir and the mode of transmission.

## 2 Materials and methods

Fresh fecal samples of 42 pre-weaned (0–2 months old) and 78 post-weaned (3–12 months old) bovine calves were collected from various dairy farms (Table 1). The samples were collected directly from the animals in sterile container by trained sample collector and immediately send to the lab and processed. Fresh fecal samples were collected from 26 humans working at the dairy farms, as directed by physicians (Table 1). The scoring system for clinical signs and diarrhea are shown in Supplementary Data 1. Additionally, 15 water samples were collected from the 15 different waterbodies located near these farms and used for the disposal of farm waste. The water samples were immediately sent to the lab for processing. Both farm animals and farm workers have regular access to those water bodies. All farms and the water bodies used for collecting samples are located in and around Haripal (22°81′N, 88°10′E), Bhadreshwar (22°82′ N, 88°35′E), Gurap (23°03′ N, 88°12′E), and Dankuni (22°66′N, 88°29′E) in West Bengal, India (Table 1). All samples were collected and transferred at 4 °C and were processed within 24–48 h.

The study received ethical clearance from National Institute of Cholera and Enteric Diseases Institutional Ethical Committee. Informed consents were obtained from the individuals (in case of children, consents were obtained from their parents). The children were from family members of farm workers. They stayed with their parents in the dairy farms and were in close contact with the animals, and because some children had diarrhea and intestinal symptoms, they were included in the study. Fecal samples were first concentrated by a previously described method (Khan et al., 2010). In case of water samples, 1–2 l of each sample was first filtered through 0.45 μm pore sized Millipore cellulose acetate membrane filter to retain any oocyst on the surface (MilliporeSigma, US). The filter paper was then rinsed with distilled water and the solution centrifuged at 400 x*g* for 10 min. The pellet was resuspended in 500 μl of distilled water and used in further procedures.

**Table 1:** Detailed information of *Cryptosporidium* infection identified in our study regions.

Area	Pre-weaned calves (0–3 months)		Post-weaned calves (3–12 months)		Human (dairy farm workers)		Water bodies		Species diversity				
	Sample tested	Positive	Sample tested	Positive	Sample tested	Positive	Sample tested	Positive	*C. parvum*	*C. ryanae*	*C. bovis*	*C. andersoni*	Total
Haripal (22°81′,88°10′ E)	11	2	25	9	8	1	4	1	4	5	3	1	13
Bhadreswar (22°82′,88°35′ E)	14	1	22	6	10	3	4	0	2	4	3	1	10
Gurap (23°03′,88°12′ E)	13	0	20	8	6	1	3	0	3	4	2	0	9
Dankuni (22°66′,88°29′ E)	4	0	11	1	2	0	4	0	0	1	0	0	1
Total	42	3 (7.1%)	78	24 (30.8%)	26	5 (19.2%)	15	1 (6.7%)	9	14	8	2	33

All study samples were initially screened by conventional microscopy followed by enzyme linked immunosorbent assay (ELISA) and PCR. Acid fast staining of all samples were performed according to CDC laboratory diagnosis protocol (http://www.dpd.cdc.gov/dpdx/HTML/DiagnosticProcedures.html) and examined by light microscopy under 100× oil immersion objective for the presence of oocysts of *Cryptosporidium* spp. The oocysts appeared light pink to red under microscope with size varying from 4 to 6 μm. All the study samples were further screened by antigen capture ELISA (CRYPTOSPORIDIUM II, TECHLAB, USA). The positive samples generated pale to strong yellow colour in wells (≥0.150 OD_450_) according to the load of oocysts in each sample.

Genomic DNA was isolated from all ELISA positive clinical samples using QIAmp DNA Stool Mini Kit (QIAGEN, USA) according to the manufacturer's protocol. Partial amplification of genetic markers (18SrRNA gene and hsp70 gene) was performed using specific primer pairs (Supplementary Data 2). In all cases the PCR reaction was carried out in 50 μl reaction volume containing approximately 0.4 μg and 0.1 μg of template DNA for primary and nested PCR respectively, 10 pM of each primer, 1.5 mM MgCl_2_, 1 μg of Bovine Serum Albumin (SIGMA, USA), 200 μM dNTP and 2.5 U of High Fidelity Taq DNA polymerase (ROCHE, Germany) with the reaction parameters as initial denaturation for 15 min or 4 min (Primary and Nested respectively) at 95 °C. This was followed by 30 cycles of denaturation at 94 °C for 30 s, annealing at 55 °C (18srRNA) and 57 °C (*hsp70*) for 30 s, extension at 72 °C for 1 min; further followed by the final extension for 10 min at 72 °C (Khan et al., 2010). The nested PCR products were separated by electrophoresis on 1.5% agarose gels (SIGMA, USA) according to their sizes. PCR products of the expected sizes were extracted from gels and purified (ROCHE, Germany). Purified PCR products were then sequenced directly with specific primers (marked with ^a^ in Supplementary Data 2) using the ‘BigDye Terminator V3.1 cycle sequencing kit’ (APPLIED BIOSYSTEMS, USA) as per the manufacturer's protocol. The labeled DNA fragments were further purified by sodium acetate and ethanol precipitation. The sequencing was carried out in an ABI 310 PRISM Automated Genetic Analyzer. Accuracy of the sequencing data was confirmed by bi-directional sequencing. The accuracy of the sequence data was also verified by repeat sequence with fresh PCR product. The nested PCR products were also subjected to restriction fragment length polymorphism (RFLP) analysis using restriction enzyme *Ssp*I according to the previously described protocol (Xiao et al., 1999).

Nucleotide sequences of each target locus (*18SrRNA* and *hsp70*) obtained from our study isolates, were aligned with reference sequences of corresponding locus (downloaded from the NCBI GenBank database) using the ClustalW multiple alignment program of MEGA version 7 software (Kumar et al., 2016). The variable sequences of each target locus (in respect to the reference sequences) were submitted to the NCBI GenBank database with accession numbers KJ584905 - KJ584913, MK947923- MK947931. Separate phylogenetic tree of each target locus (*18SrRNA* and *hsp70*) was constructed by MEGA version 7 software using a maximum likelihood matrix algorithm and bootstrap values to estimate confidence intervals (Kumar et al., 2016). Intragenic LD and number of recombination events at 18SrRNA locus of our study isolates were assessed by using DnaSP version 5.10.01 software (www.ub.es/dnasp/). Associations of *Cryptosporidium* infection with age, sex and clinical symptoms of infected animals were evaluated by Epi-Info version 3.5.4 software (Dean et al., 2011).

## 3 Results and discussion

The overall incidence of *Cryptosporidium* infection was found to be 22% in calves and 19% among dairy farm workers (Table 1), which is relatively high compared to the previous report from West Bengal, India (Khan et al., 2010). The results of analyses by microscopy, ELISA and PCR on all samples are summarized in Supplementary Data 3. “Cryptosporidium positive” samples indicate only those samples which were positive by both ELISA and PCR. Association analysis has revealed that *Cryptosporidium* infection is significantly associated with age, sex and clinical symptoms of infected animals. Infection showed a significant positive association with female calves (co-efficient value = 0.228, p value = 0.0055) aged between 3 and 12 months (co-efficient value = 0.269, p value = 0.044), but showed a significant negative association with male calves (co-efficient value = −0.228, p value = 0.0055) aged below 1 month (co-efficient value = −0.269, p value = 0.044) (Supplementary Data 4). Similar findings have been reported in a previous study from India (Maurya et al., 2013). Moreover, the analysis also showed a highly significant positive association between infection and diarrhea (co-efficient value = 0.989, p value = 0.000000), but a strong negative association with asymptomatic outcome (co-efficient value = −0.989, p value = 0.000000) in calves (Supplementary Data 4). This observation was also consistent with the previous report from India (Maurya et al., 2013).

**Fig. 1:**
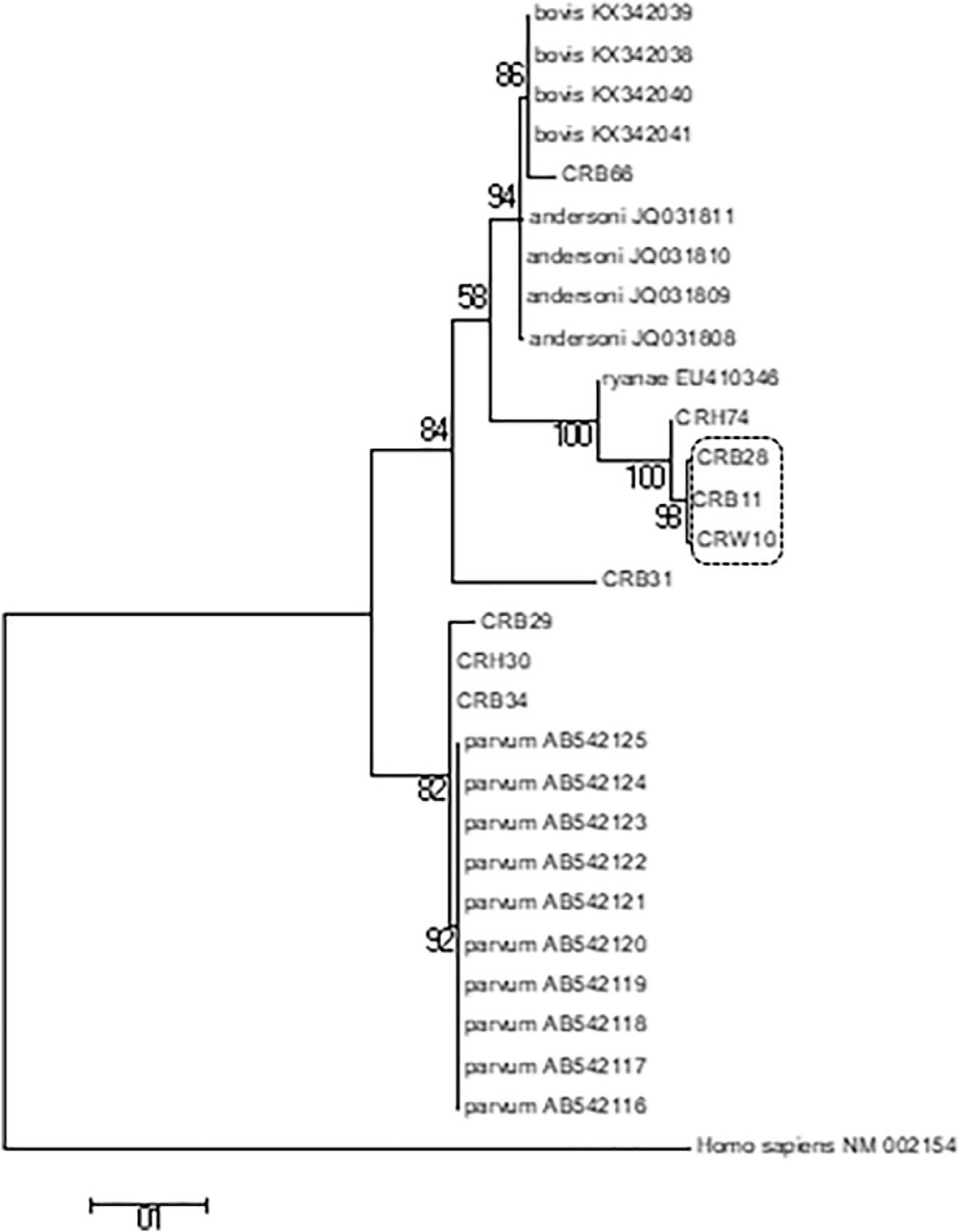
Phylogenetic analysis of our study isolates based on *hsp70* gene. Previously published *hsp70* sequences from *C. parvum, C.ryanae C. andersoni and C. bovis* were aligned with the representative sequences of our study isolates (a total of 9 sequences) using the ClustalW multiple alignment program of MEGA version 7 software. The phylogenetic tree was constructed from this alignment using maximum likelihood matrix algorithm. The bootstrap values were also analyzed to estimate confidence intervals. The study isolates from bovine and water samples formed a distinct cluster with high bootstrap value, which was marked with “dotted block”.

Analysis of RFLP and DNA sequencing based on 18SrRNA revealed a considerable amount of species diversity among *Cryptosporidium* isolates from our study region. A total of 4 *Cryptosporidium* species (*C. parvum*, *C. ryanae*, *C. bovis* and *C. andersoni*) among 9 study isolates has been identified in RFLP analysis (Supplementary Data 5, Table 1). This was further supported by Phylogenetic analysis based on *18SrRNA*, which has identified four distinct clusters, each of which representing a single or a set of species (i.e. clusters of *C. parvum*, *C. ryanae*, *C. bovis* and *C. andersoni*) (Supplementary Data 5). Moreover, our study isolates were genetically distinct compare to previously reported sequences as they formed separate cluster in phylogenetic tree with bootstrap values (Supplementary Data 5). This observation was verified by further analysis of our study isolates using *hsp70* marker to verify this observation. The sequences obtained were then compared with the previously published *hsp70* sequence (available in the NCBI database) using MEGA version 7.0 software (Kumar et al., 2016). A maximum likelihood (ML) was constructed using the same software (Fig. 1), which also supports the finding of our analysis based on *18SrRNA*. Furthermore, *C. ryanae* isolates from water samples (CRW10) and calf fecal samples (CRB11 and CRB28) form a distinct cluster with high bootstrap value (marked by “dotted block”) (Fig. 1), indicating the possibility of water contamination with *Cryptosporidium* from dairy cattle. *Cryptosporidium parvum* isolates from human (CRH30) and calves (CRB34) samples are also placed in the same position in the phylogenetic tree (Fig. 1), providing evidence in support of zoonotic transmission.

*Cryptosporidium parvum* was previously reported as the most prevalent *Cryptosporidium* species in India (Khan et al., 2010). However, *C. ryanae* which was previously identified in deer (Fayer et al., 2008) showed the highest abundance in our study region (Table 1). Simultaneous detection of *C. bovis* (Fayer et al., 2005) and *C. andersoni* (Zhao et al., 2014) together with *C. parvum* (Table 1) may indicate the possibility of cross contamination and easy transmission of this parasite. As an apicomplexan parasite, the life cycle of *Cryptosporidium* spp. has a sexual phase, during which sexual recombination can occur between genetically distinct strains (Li et al., 2013). Genetic recombination has been reported to be responsible for the emergence and spread of virulent subtypes of *Cryptosporidium* sp. (Li et al., 2013). Population genetics is an efficient method that has been employed to identify genetic recombination among the natural population of *C. hominis* (Li et al., 2013) and *C. parvum* (Feng et al., 2013). However, such evidence was lacking for *C. andersoni* (Zhao et al., 2014), *C. bovis* (Fayer et al., 2005) and *C. ryanae* (Fayer et al., 2008)*.* We identified *C ryanae* as the most prevalent infecting species in our study region and the majority of the infected animals (calves) and human (farm workers) had diarrheal symptoms (unpublished data), indicating virulence of the infecting species. We therefore investigated the possibility of genetic recombination among infecting *Cryptosporidium* sp. using LD analysis. Intragenic LD between pairs of polymorphic sites at 18SrRNA locus of our study isolates was also evaluated. The result indicates the presence of incomplete LD value (Y = 0.9592 + 0.1177×, where Y is the LD value and X is the nucleotide distance in kilobases) only within *C. ryanae* among four *Cryptosporidium* species, identified in our study (Supplementary Data 6). Among 55 pairwise comparisons, 10 were significant by the Chi-square test (Supplementary Data 6). Intragenic recombination analysis at 18SrRNA locus has also identified a minimum of 1 potential recombination events only within *C. ryanae* population (Supplementary Data 6). The incomplete LD value with potential recombination events, which was identified for the first time within our *C. ryanae* population, is a remarkable finding. This particular characteristic of the parasite is quite significant, since sexual reproduction can exchange genes, responsible for drug resistance and pathogenic behavior of parasite (Weedall and Hall, 2011).

In conclusion, our data reveal an increased occurrence of *Cryptosporidium* infection in our study region, compared to the findings of previous study (Khan et al., 2010), which may indicate a higher public health risk. The risk group associated with *Cryptosporidium* infection has also been identified in our study region. The infection was significantly present among female calves (co-efficient value = 0.228, p value = 0.0055) aged between 3 and 12 months (co-efficient value = 0.269, p value = 0.044). The infection also showed significant association with diarrheal outcome in calves (co-efficient value = 0.989, p value = 0.000000). *Cryptosporidium parvum* was previously reported as the most prevalent *Cryptosporidium* species in India (Khan et al., 2010). However, the present study has identified *C. ryanae* rather than *C. parvum* as the most prevalent infecting species in the study region. Evidence for genetic recombination has been identified for the first time in *C. ryanae*, which was previously reported to have a clonal population structure. Finally, the high rate of *Cryptosporidium* infection among calves, and our findings of oocyst contaminated water and zoonotic transmission, suggest a serious public health risk for the dairy farm workers and villagers living in close proximity.

The following are the supplementary data related to this article.

Supplementary Data 1 Table showing the scoring system for clinical signs and diarrhea Supplementary Data 1

Supplementary Data 2 List of gene specific PCR primers, used in the study. Supplementary Data 2

Supplementary Data 3 Table showing the summary results of clinical samples analyzed in the study Supplementary Data 3

Supplementary Data 4 Analysis by Epi-Info version 3.5.4 software revealed significant association of Cryptosporidium infection with age, sex and clinical symptoms of infected animals. Supplementary Data 4

**Supplementary Data 5:**
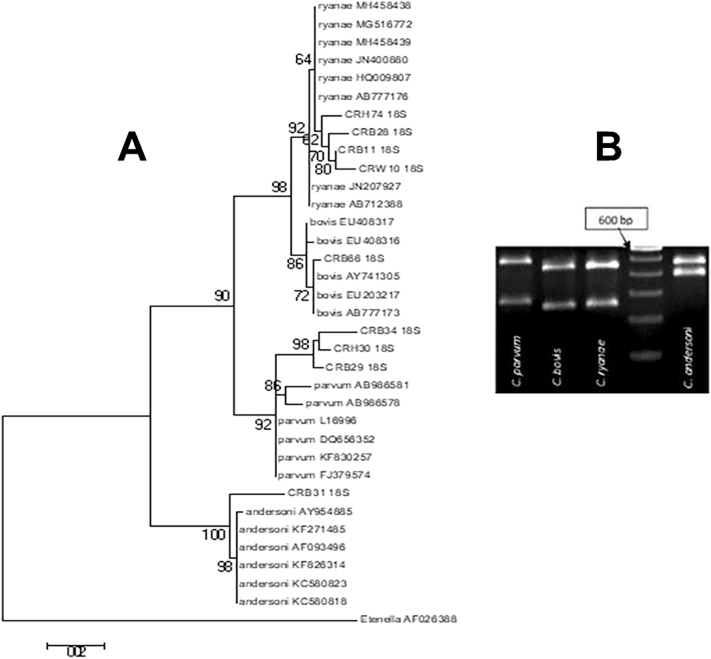
Analysis of genetic diversity among *Cryptosporidium* isolates based on *18SrRNA* gene: A. Phylogenetic tree. Previously published *18SrRNA* sequences of *Cryptosporidium* species were aligned with the representative sequences of our study isolates (a total of 9 sequences) using the ClustalW multiple alignment program of MEGA version 7 software. The phylogenetic tree was constructed from this alignment using maximum likelihood matrix algorithm. The bootstrap values were also analyzed to estimate confidence intervals. B. Restriction fragment length polymorphism (RFLP) profiles of *Cryptosporidium* isolates on an agarose gel

Supplementary Data 6 Intragenic linkage disequilibrium (LD) and recombination analysis at 18SrRNA locus of our study isolates using DnaSP version 5.10.01software. Supplementary Data 6

## Declaration of Competing Interest

The authors declare that they have no known competing financial interests or personal relationships that could have appeared to influence the work reported in this paper.

## References

[bb0005] Dean A.G., Arner T.G., Sunki G.G., Friedman R., Lantinga M., Sangam S. (2011). Epi Info™, A Database and Statistics Program for Public Health Professionals.

[bb0010] Fayer R., Santín M., Xiao L. (2005). *Cryptosporidium bovis* n. sp. (Apicomplexa: Cryptosporidiidae) in cattle (Bos taurus). J. Parasitol..

[bb0015] Fayer R., Santín M., Trout J.M. (2008). *Cryptosporidium ryanae* n. sp. (Apicomplexa: Cryptosporidiidae) in cattle (Bos taurus). Vet. Parasitol..

[bb0020] Feng Y., Torres E., Li N., Wang L., Bowman D., Xiao L. (2013). Population genetic characterisation of dominant *Cryptosporidium parvum* subtype IIaA15G2R1. Int. J. Parasitol..

[bb0025] Jex A.R., Smith H.V., Nolan M.J., Campbell B.E., Young N.D., Cantacessi C. (2011). Cryptic parasite revealed improved prospects for treatment and control of human cryptosporidiosis through advanced technologies. Adv. Parasitol..

[bb0030] Khan S.M., Debnath C., Pramanik A.K., Xiao L., Nozaki T., Ganguly S. (2010). Molecular characterization and assessment of zoonotic transmission of *Cryptosporidium* from dairy cattle in West Bengal, India. Vet. Parasitol..

[bb0035] Kumar S., Stecher G., Tamura K. (2016). MEGA7: molecular evolutionary genetics analysis version 7.0 for bigger datasets. Mol. Biol. Evol..

[bb0040] Kurniawan A., Dwintasari S.W., Connelly L., Nichols R.A., Yunihastuti E., Karyadi T. (2013). *Cryptosporidium* species from human immunodeficiency-infected patients with chronic diarrhea in Jakarta, Indonesia. Ann. Epidemiol..

[bb0045] Li N., Xiao L., Cama V.A., Ortega Y., Gilman R.H., Guo M. (2013). Genetic recombination and *Cryptosporidium hominis* virulent subtype IbA10G2. Emerg. Infect. Dis..

[bb0050] Maurya P.S., Rakesh R.L., Pradeep B., Kumar S., Kundu K., Garg R. (2013). Prevalence and risk factors associated with *Cryptosporidium spp.* infection in young domestic livestock in India. Trop. Anim. Health Prod..

[bb0055] Steeb S., Hagedorn H.J., Krone J.R. (1987). Cryptosporidiosis in immunocompetent patients. Epidemiology and clinical picture. Dtsch. Med. Wochenschr..

[bb0060] Weedall G.D., Hall N. (2011). Evolutionary genomics of *Entamoeba*. Resmic..

[bb0065] Xiao L. (2010). Molecular epidemiology of cryptosporidiosis: an update. Exp. Parasitol..

[bb0070] Xiao L., Escalante L., Yang C., Sulaiman I., Escalante A.A., Montali R.J. (1999). Phylogenetic analysis of *Cryptosporidium* parasites based on the small-subunit rRNA gene locus. Appl. Environ. Microbiol..

[bb0075] Zhao W., Wang R., Zhang W., Liu A., Cao J, Shen Y, et al., 2014. MLST Subtypes and Population Genetic Structure of *Cryptosporidium andersoni* from Dairy Cattle and Beef Cattle in Northeastern China's Heilongjiang Province, PLoS One. vol. 7, e102006. 10.1371/journal.pone.0102006 PMC4084942 24999982

