# Supplementary material for: Genetic characterization reveals evidence for an association between water contamination and zoonotic transmission of a Cryptosporidium sp. from dairy cattle in West Bengal, India
Source: Food Waterborne Parasitol. 2019 Aug 22;17:e00064. doi: 10.1016/j.fawpar.2019.e00064 (PMC7034051; doi:10.1016/j.fawpar.2019.e00064)
Supplement: Supplementary Data 1 — Table showing the scoring system for clinical signs and diarrhea [file mmc1.docx]

| Diseases Indicator | Category | Score |
| --- | --- | --- |
| Dehydration | Mild | 1 |
|  | Moderate | 2 |
|  | Severe | 3 |
| Fever | No | 0 |
|  | Yes | 1 |
| Vomiting | No | 0 |
|  | Yes | 1 |
| Duration of Diarrhea (days) | 1-4 | 1 |
|  | 5-7 | 2 |
|  | ≥8 | 3 |
