# Supplementary material for: Genetic characterization reveals evidence for an association between water contamination and zoonotic transmission of a Cryptosporidium sp. from dairy cattle in West Bengal, India
Source: Food Waterborne Parasitol. 2019 Aug 22;17:e00064. doi: 10.1016/j.fawpar.2019.e00064 (PMC7034051; doi:10.1016/j.fawpar.2019.e00064)
Supplement: Supplementary Data 2 — List of gene specific PCR primers, used in the study. [file mmc2.docx]

| Gene name | PCR round | Primer name | Primer sequence (5’ to 3’) |
| --- | --- | --- | --- |
| ***18srRNA*** | Primary | Cr18S_F1 | TTCTAGAGCTAATACATGCG |
|  |  | Cr18S_R1 | CCCATTTCCTTCGAAACAGGA |
|  | Nested | Cr18S_F2**^a^** | TAGGAGTAGGAGCTTCACC |
|  |  | Cr18S_R2**^a^** | AAGGAGTAAGGAACAACCTCCA |
| ***hsp70*** | Primary | Crhsp70_F1 | GTGATATGACTCACTGGCCATTTA |
|  |  | Crhsp70_R1 | TTGCTTTGCATGGCTCTTTACCGTT |
|  | Nested | Crhsp70_F2**^a^** | ATTGAAGATGGTATTTTTGAAG |
|  |  | Crhsp70_R2**^a^** | CAACCAATACAACATCATGTACAG |

**F -** Forward primer (**F1**- for Primary PCR, **F2**- for Nested PCR), **R**- Reverse primer (**R1**- for Primary PCR, **R2**- for Nested PCR). ^a^ Gene specific primer pairs used for sequencing of amplified PCR products.
