# Supplementary material for: Genetic characterization reveals evidence for an association between water contamination and zoonotic transmission of a Cryptosporidium sp. from dairy cattle in West Bengal, India
Source: Food Waterborne Parasitol. 2019 Aug 22;17:e00064. doi: 10.1016/j.fawpar.2019.e00064 (PMC7034051; doi:10.1016/j.fawpar.2019.e00064)
Supplement: Supplementary Data 3 — Table showing the summary results of clinical samples analyzed in the study [file mmc3.docx]

| **SL.**  **No** | **SAMPLE** | **SOURCE** | **RESULT** | | |
| --- | --- | --- | --- | --- | --- |
|  |  |  | **Microscopy** | **ELISA** | **PCR** |
| 1 | CRBP01 | Pre-weaned calves | **-** | **-** | **-** |
| 2 | CRBP02 |  | **-** | **-** | **-** |
| 3 | CRBP03 |  | **-** | **-** | **-** |
| 4 | CRBP04 |  | - | - | - |
| 5 | CRBP05 |  | **-** | **-** | **-** |
| 6 | CRBP06 |  | **-** | **-** | **-** |
| 7 | CRBP07 |  | - | + | + |
| 8 | CRBP08 |  | **-** | **-** | **-** |
| 9 | CRBP09 |  | **-** | **-** | **-** |
| 10 | CRBP10 |  | **-** | **-** | **-** |
| 11 | CRBP11 |  | **-** | **-** | **-** |
| 12 | CRBP12 |  | **-** | **-** | **-** |
| 13 | CRBP13 |  | **-** | **-** | **-** |
| 14 | CRBP14 |  | **-** | **-** | **-** |
| 15 | CRBP15 |  | **-** | **-** | **-** |
| 16 | CRBP16 |  | **-** | + | + |
| 17 | CRBP17 |  | **-** | **-** | **-** |
| 18 | CRBP18 |  | **-** | **-** | **-** |
| 19 | CRBP19 |  | **-** | **-** | **-** |
| 20 | CRBP20 |  | **-** | **-** | **-** |
| 21 | CRBP21 |  | **-** | **-** | **-** |
| 22 | CRBP22 |  | **-** | **-** | **-** |
| 23 | CRBP23 |  | **-** | **-** | **-** |
| 24 | CRBP24 |  | **-** | **-** | **-** |
| 25 | CRBP25 |  | **-** | **-** | **-** |
| 26 | CRBP26 |  | **-** | **-** | **-** |
| 27 | CRBP27 |  | **-** | + | + |
| 28 | CRBP28 |  | **-** | **-** | **-** |
| 29 | CRBP29 |  | **-** | **-** | **-** |
| 30 | CRBP30 |  | **-** | **-** | **-** |
| 31 | CRBP31 |  | **-** | **-** | - |
| 32 | CRBP32 |  | **-** | **-** | **-** |
| 33 | CRBP33 |  | **-** | **-** | **-** |
| 34 | CRBP34 |  | **-** | **-** | **-** |
| 35 | CRBP35 |  | **-** | **-** | **-** |
| 36 | CRBP36 |  | **-** | **-** | **-** |
| 37 | CRBP37 |  | **-** | **-** | **-** |
| 38 | CRBP38 |  | **-** | **-** | **-** |
| 39 | CRBP39 |  | **-** | **-** | **-** |
| 40 | CRBP40 |  | **-** | **-** | **-** |
| 41 | CRBP41 |  | **-** | **-** | **-** |
| 42 | CRBP42 |  | **-** | **-** | **-** |
| 43 | CRB01 | Post-weaned calves | **-** | **-** | **-** |
| 44 | CRB02 |  | **-** | **-** | **-** |
| 45 | CRB03 |  | - | + | + |
| 46 | CRB04 |  | **-** | **-** | **-** |
| 47 | CRB05 |  | **-** | **-** | **-** |
| 48 | CRB06 |  | **-** | **-** | **-** |
| 49 | CRB07 |  | - | + | + |
| 50 | CRB08 |  | **-** | **-** | **-** |
| 51 | CRB09 |  | **-** | **-** | **-** |
| 52 | CRB10 |  | - | + | + |
| 53 | **CRB11** |  | **+** | **+** | **+** |
| 54 | CRB12 |  | - | + | + |
| 55 | CRB13 |  | **-** | **-** | **-** |
| 56 | CRB14 |  | **-** | **-** | **-** |
| 57 | CRB15 |  | **-** | **-** | **-** |
| 58 | CRB16 |  | - | + | + |
| 59 | CRB17 |  | **-** | **-** | **-** |
| 60 | CRB18 |  | **-** | **-** | **-** |
| 61 | CRB19 |  | **-** | **-** | **-** |
| 62 | CRB20 |  | **-** | **-** | **-** |
| 63 | CRB21 |  | **-** | **-** | **-** |
| 64 | CRB22 |  | - | + | + |
| 65 | CRB23 |  | **-** | **-** | **-** |
| 66 | CRB24 |  | **-** | **-** | **-** |
| 67 | CRB25 |  | **-** | **-** | **-** |
| 68 | CRB26 |  | **-** | **-** | **-** |
| 69 | CRB27 |  | - | + | + |
| 70 | **CRB28** |  | **+** | **+** | **+** |
| 71 | **CRB29** |  | **+** | **+** | **+** |
| 72 | CRB30 |  | **-** | **-** | **-** |
| 73 | **CRB31** |  | **+** | **+** | **+** |
| 74 | CRB32 |  | **-** | **-** | **-** |
| 75 | CRB33 |  | - | + | + |
| 76 | **CRB34** |  | **+** | **+** | **+** |
| 77 | CRB35 |  | - | - | - |
| 78 | CRB36 |  | - | - | - |
| 79 | CRB37 |  | - | + | + |
| 80 | CRB38 |  | - | - | - |
| 81 | CRB39 |  | - | - | - |
| 82 | CRB40 |  | **-** | **-** | **-** |
| 83 | CRB41 |  | **-** | **-** | **-** |
| 84 | CRB42 |  | **-** | **-** | **-** |
| 85 | CRB43 |  | - | + | + |
| 86 | CRB44 |  | **-** | **-** | **-** |
| 87 | CRB45 |  | **-** | **-** | **-** |
| 88 | CRB46 |  | - | + | + |
| 89 | CRB47 |  | - | - | - |
| 90 | CRB48 |  | - | - | - |
| 91 | CRB49 |  | - | + | + |
| 92 | CRB50 |  | - | + | + |
| 93 | CRB51 |  | - | - | - |
| 94 | CRB52 |  | - | - | - |
| 95 | CRB53 |  | - | + | + |
| 96 | CRB54 |  | **-** | **-** | **-** |
| 97 | CRB55 |  | **-** | **-** | **-** |
| 98 | CRB56 |  | **-** | **-** | **-** |
| 99 | CRB57 |  | **-** | **-** | **-** |
| 100 | CRB58 |  | - | + | + |
| 101 | CRB59 |  | - | + | + |
| 102 | CRB60 |  | **-** | **-** | **-** |
| 103 | CRB61 |  | **-** | **-** | **-** |
| 104 | CRB62 |  | - | + | + |
| 105 | CRB63 |  | **-** | **-** | **-** |
| 106 | CRB64 |  | **-** | **-** | **-** |
| 107 | CRB65 |  | **-** | **-** | **-** |
| 108 | **CRB66** |  | **+** | **+** | **+** |
| 109 | CRB67 |  | **-** | **-** | **-** |
| 110 | CRB68 |  | **-** | **-** | **-** |
| 111 | CRB69 |  | **-** | **-** | **-** |
| 112 | CRB70 |  | **-** | **-** | **-** |
| 113 | CRB71 |  | - | + | + |
| 114 | CRB72 |  | **-** | **-** | **-** |
| 115 | CRB73 |  | **-** | **-** | **-** |
| 116 | CRB74 |  | **-** | **-** | **-** |
| 117 | CRB75 |  | **-** | **-** | **-** |
| 118 | CRB76 |  | **-** | **-** | **-** |
| 119 | CRB77 |  | **-** | **-** | **-** |
| 120 | CRB78 |  | **-** | **-** | **-** |
| 121 | CRH01 | Human  (Dairy farm workers) | **-** | **-** | **-** |
| 122 | CRH08 |  | **-** | **-** | **-** |
| 123 | CRH15 |  | **-** | **-** | **-** |
| 124 | CRH21 |  | **-** | **-** | **-** |
| 125 | CRH23 |  | **-** | **-** | **-** |
| 126 | CRH27 |  | **-** | **-** | **-** |
| 127 | **CRH30** |  | **+** | **+** | **+** |
| 128 | CRH34 |  | **-** | **-** | **-** |
| 129 | CRH39 |  | **-** | **-** | **-** |
| 130 | CRH46 |  | **-** | **-** | **-** |
| 131 | CRH51 |  | **-** | **-** | **-** |
| 132 | CRH56 |  | - | + | + |
| 133 | CRH59 |  | - | - | - |
| 134 | CRH63 |  | - | + | + |
| 135 | CRH65 |  | **-** | **-** | **-** |
| 136 | CRH68 |  | **-** | **-** | **-** |
| 137 | **CRH74** |  | **+** | **+** | **+** |
| 138 | CRH77 |  | **-** | **-** | **-** |
| 139 | CRH82 |  | **-** | **-** | **-** |
| 140 | CRH87 |  | - | + | + |
| 141 | CRH89 |  | **-** | **-** | **-** |
| 142 | CRH92 |  | **-** | **-** | **-** |
| 143 | CRH95 |  | **-** | **-** | **-** |
| 144 | CRH101 |  | **-** | **-** | **-** |
| 145 | CRH107 |  | **-** | **-** | **-** |
| 146 | CRH112 |  | **-** | **-** | **-** |
| 147 | CRW01 | Water Bodies | **-** | **-** | **-** |
| 148 | CRW02 |  | **-** | **-** | **-** |
| 149 | CRW03 |  | **-** | **-** | **-** |
| 150 | CRW04 |  | **-** | **-** | **-** |
| 151 | CRW05 |  | **-** | **-** | **-** |
| 152 | CRW06 |  | **-** | **-** | **-** |
| 153 | CRW07 |  | **-** | **-** | **-** |
| 154 | CRW08 |  | **-** | **-** | **-** |
| 155 | CRW09 |  | **-** | **-** | **-** |
| 156 | **CRW10** |  | **+** | **+** | **+** |
| 157 | CRW11 |  | **-** | **-** | **-** |
| 158 | CRW12 |  | **-** | **-** | **-** |
| 159 | CRW13 |  | **-** | **-** | **-** |
| 160 | CRW14 |  | **-** | **-** | **-** |
| 161 | CRW15 |  | **-** | **-** | **-** |

“+” indicates positive, while “-”indicates negative result in corresponding test.
