# Supplementary material for: Genetic characterization reveals evidence for an association between water contamination and zoonotic transmission of a Cryptosporidium sp. from dairy cattle in West Bengal, India
Source: Food Waterborne Parasitol. 2019 Aug 22;17:e00064. doi: 10.1016/j.fawpar.2019.e00064 (PMC7034051; doi:10.1016/j.fawpar.2019.e00064)
Supplement: Supplementary Data 4 — Analysis by Epi-Info version 3.5.4 software revealed significant association of Cryptosporidium infection with age, sex and clinical symptoms of infected animals. [file mmc4.docx]

**Epi Info**

[Results Library](file:///G:\\Epi_Info\\IResults.htm)

| *Current View:* | **C:\Users\user\Desktop\Crypto paper\LAKSHMI\CALF SAMPLES edit.xls:Sheet1$** | | | | |
| --- | --- | --- | --- | --- | --- |
| *Record Count:* | **120** |  |  | *Date:* | **1/26/2015 10:41:54 PM** |

**REGRESS E (Cryptosporidium infection) = MALE**

[Next Procedure](file:///G:\Epi_Info\OUT108.htm#Contents1_2)

**Linear Regression**

| **Variable** | **Coefficient** | **Std Error** | **F-test** | **P-Value** |
| --- | --- | --- | --- | --- |
| **MALE** | -0.228 | 0.081 | 7.9696 | 0.005592 |
| **CONSTANT** | 0.342 | 0.049 | 48.7336 | 0.000000 |

| **Correlation Coefficient: r^2=** | 0.06 |
| --- | --- |

| **Source** | **df** | **Sum of Squares** | **Mean Square** | **F-statistic** |
| --- | --- | --- | --- | --- |
| **Regression** | 1 | 1.455 | 1.455 | 7.970 |
| **Residuals** | 118 | 21.537 | 0.183 |  |
| **Total** | 119 | 22.992 |  |  |

[Previous Dataset](file:///G:\\Epi_Info\\OUT108.htm#Results1)[Results Library](file:///G:\Epi_Info\IResults.htm)

**REGRESS E (Cryptosporidium infection) = FEMALE**

[Next Procedure](file:///G:\Epi_Info\OUT108.htm#Contents2_2)

**Linear Regression**

| **Variable** | **Coefficient** | **Std Error** | **F-test** | **P-Value** |
| --- | --- | --- | --- | --- |
| **FEMALE** | 0.228 | 0.081 | 7.9696 | 0.005592 |
| **CONSTANT** | 0.114 | 0.064 | 3.1130 | 0.080278 |

| **Correlation Coefficient: r^2=** | 0.06 |
| --- | --- |

| **Source** | **df** | **Sum of Squares** | **Mean Square** | **F-statistic** |
| --- | --- | --- | --- | --- |
| **Regression** | 1 | 1.455 | 1.455 | 7.970 |
| **Residuals** | 118 | 21.537 | 0.183 |  |
| **Total** | 119 | 22.992 |  |  |

[Previous Dataset](file:///G:\\Epi_Info\\OUT108.htm#Results2)[Results Library](file:///G:\Epi_Info\IResults.htm)

**REGRESS E (Cryptosporidium infection) = A (3-12 months)**

[Next Procedure](file:///G:\Epi_Info\OUT108.htm#Contents3_2)

**Linear Regression**

| **Variable** | **Coefficient** | **Std Error** | **F-test** | **P-Value** |
| --- | --- | --- | --- | --- |
| **A** | 0.269 | 0.132 | 4.1366 | 0.044226 |
| **CONSTANT** | 0.231 | 0.042 | 30.7420 | 0.000000 |

| **Correlation Coefficient: r^2=** | 0.03 |
| --- | --- |

| **Source** | **df** | **Sum of Squares** | **Mean Square** | **F-statistic** |
| --- | --- | --- | --- | --- |
| **Regression** | 1 | 0.779 | 0.779 | 4.137 |
| **Residuals** | 118 | 22.213 | 0.188 |  |
| **Total** | 119 | 22.992 |  |  |

[Previous Dataset](file:///G:\\Epi_Info\\OUT108.htm#Results3)[Results Library](file:///G:\Epi_Info\IResults.htm)

**REGRESS E (Cryptosporidium infection) = B (0-3 months)**

[Next Procedure](file:///G:\Epi_Info\OUT108.htm#Contents4_2)

**Linear Regression**

| **Variable** | **Coefficient** | **Std Error** | **F-test** | **P-Value** |
| --- | --- | --- | --- | --- |
| **B** | -0.269 | 0.132 | 4.1366 | 0.044226 |
| **CONSTANT** | 0.500 | 0.125 | 15.9366 | 0.000115 |

| **Correlation Coefficient: r^2=** | 0.03 |
| --- | --- |

| **Source** | **df** | **Sum of Squares** | **Mean Square** | **F-statistic** |
| --- | --- | --- | --- | --- |
| **Regression** | 1 | 0.779 | 0.779 | 4.137 |
| **Residuals** | 118 | 22.213 | 0.188 |  |
| **Total** | 119 | 22.992 |  |  |

[Previous Dataset](file:///G:\Epi_Info\OUT108.htm#Results4)[Results Library](file:///G:\Epi_Info\IResults.htm)

**REGRESS E (Cryptosporidium infection) = ND (Asymtomatic outcome)**

[Next Procedure](file:///G:\Epi_Info\OUT109.htm#Contents1_2)

**Linear Regression**

| **Variable** | **Coefficient** | **Std Error** | **F-test** | **P-Value** |
| --- | --- | --- | --- | --- |
| **ND** | -0.989 | 0.019 | 2625.5000 | 0.000000 |
| **CONSTANT** | 1.000 | 0.017 | 3579.7753 | 0.000000 |

| **Correlation Coefficient: r^2=** | 0.96 |
| --- | --- |

| **Source** | **df** | **Sum of Squares** | **Mean Square** | **F-statistic** |
| --- | --- | --- | --- | --- |
| **Regression** | 1 | 22.003 | 22.003 | 2625.500 |
| **Residuals** | 118 | 0.989 | 0.008 |  |
| **Total** | 119 | 22.992 |  |  |

[Previous Dataset](file:///G:\Epi_Info\OUT109.htm#Results1)[Results Library](file:///G:\Epi_Info\IResults.htm)

**REGRESS E (Cryptosporidium infection) = D (Diarrheal outcome)**

[Next Procedure](file:///G:\Epi_Info\OUT109.htm#Contents2_2)

**Linear Regression**

| **Variable** | **Coefficient** | **Std Error** | **F-test** | **P-Value** |
| --- | --- | --- | --- | --- |
| **D** | 0.989 | 0.019 | 2625.5000 | 0.000000 |
| **CONSTANT** | 0.011 | 0.010 | 1.3258 | 0.251894 |

| **Correlation Coefficient: r^2=** | 0.96 |
| --- | --- |

| **Source** | **df** | **Sum of Squares** | **Mean Square** | **F-statistic** |
| --- | --- | --- | --- | --- |
| **Regression** | 1 | 22.003 | 22.003 | 2625.500 |
| **Residuals** | 118 | 0.989 | 0.008 |  |
| **Total** | 119 | 22.992 |  |  |
