# Supplementary material for: Genetic characterization reveals evidence for an association between water contamination and zoonotic transmission of a Cryptosporidium sp. from dairy cattle in West Bengal, India
Source: Food Waterborne Parasitol. 2019 Aug 22;17:e00064. doi: 10.1016/j.fawpar.2019.e00064 (PMC7034051; doi:10.1016/j.fawpar.2019.e00064)
Supplement: Supplementary Data 6 — Intragenic linkage disequilibrium (LD) and recombination analysis at 18SrRNA locus of our study isolates using DnaSP version 5.10.01software. [file mmc5.docx]

**DNA polymorphism**

Input Data File: C:\...\***C. ryanae*** fasta.fas

Number of sequences: 4 Number of sequences used: 4

Selected region: 1-453 Number of sites: 453

Total number of sites (excluding sites with gaps / missing data): 449

Number of polymorphic (segregating) sites, S: 11

Total number of mutations, Eta: 11

Number of Haplotypes, h: 4

Haplotype (gene) diversity, Hd: 1.000

Variance of Haplotype diversity: 0.03125

Standard Deviation of Haplotype diversity: 0.177

Nucleotide diversity, Pi: 0.01299

Theta (per site) from Eta: 0.01336

Theta (per site) from S, Theta-W: 0.01336

Variance of theta (no recombination): 0.0000630

Standard deviation of theta (no recombination): 0.00794

Variance of theta (free recombination): 0.0000162

Standard deviation of theta (free recombination): 0.00403

Finite Sites Model

Theta (per site) from Pi: 0.01322

Theta (per site) from S: 0.01364

Theta (per site) from Eta: 0.01358

Average number of nucleotide differences, k: 5.833

Stochastic variance of k (no recombination), Vst(k): 6.742

Sampling variance of k (no recombination), Vs(k): 5.694

Total variance of k (no recombination), V(k): 12.437

Stochastic variance of k (free recombination), Vst(k): 1.944

Sampling variance of k (free recombination), Vs(k): 1.296

Total variance of k (free recombination), V(k): 3.241

Theta (per sequence) from S, Theta-W: 6.000

Variance of theta (no recombination): 12.706

Variance of theta (free recombination): 3.273

**Linkage disequilibrium analysis**

Input Data File: C:\...\ ***C. ryanae*** fasta.fas

Number of sequences: 4 Number of sequences used: 4

Selected region: 1-453 Number of sites: 453

Total number of sites (excluding sites with gaps / missing data): 449

All Polymorphic sites were considered

Number of polymorphic sites analyzed: 11

**Number of pairwise comparisons: 55**

**Number of significant pairwise comparisons by chi-square test: 10**

Number of significant comparisons using the Bonferroni procedure: 0

Value of ZnS (Kelly 1997): 0.3434

Value of Za (Rozas et al. 2001): 0.3778

Value of ZZ (Rozas et al. 2001): 0.0343

Value of Wall's B: 0.2000

Value of Wall's Q: 0.2727

Table. * 0.01<P<0.05; ** 0.001<P<0.01; *** P<0.001

B, significant by the Bonferroni procedure

===== Regression Equation: Y = a + bX (X measured in kb) =====

|D| values: Y = 0.1069 - 0.0124X (55 points)

**|D'| values: Y = 0.9592 + 0.1177X (55 points)**

r^2 values: Y = 0.3559 - 0.0648X (55 points)

------------------------------------------------------------------------------------

Site1 Site2 Dist D D' R Chi-sq

13 50 36 -0.063 -1.000 -0.333 0.444

13 51 37 -0.125 -1.000 -0.577 1.333

13 52 38 0.188 1.000 1.000 4.000*

13 57 42 -0.063 -1.000 -0.333 0.444

13 61 46 0.125 1.000 0.577 1.333

13 95 80 -0.063 -1.000 -0.333 0.444

13 393 378 0.188 1.000 1.000 4.000*

13 416 401 -0.063 -1.000 -0.333 0.444

13 417 402 -0.063 -1.000 -0.333 0.444

13 418 403 -0.063 -1.000 -0.333 0.444

50 51 1 0.125 1.000 0.577 1.333

50 52 2 -0.063 -1.000 -0.333 0.444

50 57 6 -0.063 -1.000 -0.333 0.444

50 61 10 0.125 1.000 0.577 1.333

50 95 44 -0.063 -1.000 -0.333 0.444

50 393 342 -0.063 -1.000 -0.333 0.444

50 416 365 0.188 1.000 1.000 4.000*

50 417 366 0.188 1.000 1.000 4.000*

50 418 367 0.188 1.000 1.000 4.000*

51 52 1 -0.125 -1.000 -0.577 1.333

51 57 5 -0.125 -1.000 -0.577 1.333

51 61 9 0.000 0.000 0.000 0.000

51 95 43 -0.125 -1.000 -0.577 1.333

51 393 341 -0.125 -1.000 -0.577 1.333

51 416 364 0.125 1.000 0.577 1.333

51 417 365 0.125 1.000 0.577 1.333

51 418 366 0.125 1.000 0.577 1.333

52 57 4 -0.063 -1.000 -0.333 0.444

52 61 8 0.125 1.000 0.577 1.333

52 95 42 -0.063 -1.000 -0.333 0.444

52 393 340 0.188 1.000 1.000 4.000*

52 416 363 -0.063 -1.000 -0.333 0.444

52 417 364 -0.063 -1.000 -0.333 0.444

52 418 365 -0.063 -1.000 -0.333 0.444

57 61 4 -0.125 -1.000 -0.577 1.333

57 95 38 0.188 1.000 1.000 4.000*

57 393 336 -0.063 -1.000 -0.333 0.444

57 416 359 -0.063 -1.000 -0.333 0.444

57 417 360 -0.063 -1.000 -0.333 0.444

57 418 361 -0.063 -1.000 -0.333 0.444

61 95 34 -0.125 -1.000 -0.577 1.333

61 393 332 0.125 1.000 0.577 1.333

61 416 355 0.125 1.000 0.577 1.333

61 417 356 0.125 1.000 0.577 1.333

61 418 357 0.125 1.000 0.577 1.333

95 393 298 -0.063 -1.000 -0.333 0.444

95 416 321 -0.063 -1.000 -0.333 0.444

95 417 322 -0.063 -1.000 -0.333 0.444

95 418 323 -0.063 -1.000 -0.333 0.444

393 416 23 -0.063 -1.000 -0.333 0.444

393 417 24 -0.063 -1.000 -0.333 0.444

393 418 25 -0.063 -1.000 -0.333 0.444

416 417 1 0.188 1.000 1.000 4.000*

416 418 2 0.188 1.000 1.000 4.000*

417 418 1 0.188 1.000 1.000 4.000*

**Recombination analysis**

Input Data File: C:\...\ ***C. ryanae*** fasta.fas

Number of sequences: 4 Number of sequences used: 4

Selected region: 1-453 Number of sites: 453

Total number of sites (excluding sites with gaps / missing data): 449

Number of polymorphic (segregating) sites, S: 11

Estimate of the recombination parameter, R (or C) (Hudson 1987)

Variance of the sample distribution of Kij, Sk^2: 8.734

Estimate of Theta, per gene: 5.833

Value of the function g(C,n): 0.1800

Estimate of R, per gene: Greater than 10000

Average nucleotide distance between the most distant sites: 449.50

Minimum number of recombination events (Hudson and Kaplan 1985)

Number of pairwise comparisons analyzed: 55

Number of pairs of sites with four gametic types: 1

(51,61)

**Minimum number of recombination events, Rm: 1**

Recombination has been detected between sites:

(51,61)
